# Spatial Effects of Environmental Pollution on Healthcare Services: Evidence from China

**DOI:** 10.3390/ijerph18041784

**Published:** 2021-02-12

**Authors:** Ning Zhang, Ying Mao

**Affiliations:** 1 School of Public Policy and Administration, Xi’an Jiaotong University, Xi’an 710049, China; 2 Research Center for the Belt and Road Health Policy and Health Technology Assessment, Xi’an Jiaotong University, Xi’an 710049, China

**Keywords:** spatial effects, environmental pollution, healthcare services

## Abstract

With the rapid development of urbanization and industrialization in China, environmental issues have become an urgent problem, especially issues related to air, water, and solid-waste pollution. These pollutants pose threats to the health of the population and to that of communities and have a vicious influence on the healthcare system. Additionally, pollution also exhibits spill-over effects, which means that pollution in the local region could affect the healthcare services in a neighboring region. Therefore, it is necessary to explore the relationship between pollution and healthcare. A spatial autocorrelation analysis was conducted and spatial panel econometric models were constructed to explore the characteristics of pollution and healthcare services in China and the relationship between them using data on all 31 provinces over 12 consecutive years (2006–2017). The results showed that the utilization of healthcare services and environmental pollution were not randomly distributed; unsurprisingly, air pollution and solid-waste pollution were mainly found in parts of northern China, while water pollution was highest in southern and coastal China. In addition, environmental pollution exhibited spill-over effects on healthcare services. For example, a 1% increase in solid waste in one specific geographical unit was estimated to increase the inpatient visits per capita in adjacent counties by 0.559%. Specifically, pollution showed different degrees of influence on healthcare services, which means that the impact of environmental pollution on the number of outpatient visits is greater than on the number of inpatient visits. Our results provide the government with evidence for effectively formulating and promulgating policies, especially policies aimed at tackling spill-over effects among different regions.

## 1. Introduction

The reform and opening-up policy in China, in place for 40 years, has changed the lives of individuals from all walks of life, as well as the health status of citizens and the quality of healthcare services [1]. However, environmental pollution along with economic development contributes to the burden of disease and the shortage of healthcare services. The Global Burden of Diseases (GBD) study summarizes the influence of pollution-related factors, such as unsafe water resources, air pollution, and other environmental risks, on health [2]. For example, air pollution is ranked as the fourth greatest risk factor in DALYs and deaths; specifically, it contributed to 213 million DALYs and 6.67 million deaths in 2019 according to the Global Burden of Disease analysis conducted by the Institute for Health Metrics and Evaluation (IHME). According to 2017 data from the World Health Organization (WHO), almost one-quarter of the world’s population does not have proper access to safe drinking water or to the most basic sanitation. Sixty percent of the world’s pollution is due to a shortage of “safely managed” sanitation [3,4,5]. Sewage disposal influences people’s immediate environments and leads to water-related illnesses such as diarrhea, which kills 525,000 children under five each year. In regard to solid waste, the amount of garbage is 75% more than the combined weight of each person in the world today.

Environmental pollution also has a serious impact on daily life. Consider air pollution in China: the Chinese government has made major progress in improving air quality in recent years. However, the majority of cities still do not meet the air quality standards of the WHO. In 2019, 48 cities in mainland China were among the 100 most polluted cities in the world. Of the 400 cities in mainland China, only 2% have an average annual PM2.5 concentration lower than the World Health Organization’s guideline value (10 μg/m^3^), and 53% of the cities have an average annual PM2.5 concentration that meets China’s air quality Grade II standard (35 μg/m^3^) [6]. It is estimated that in 2017 in China, air pollution shortened the average life expectancy by 23 months, of which outdoor and indoor air pollution reduced the average life expectancy by 15 months and 8 months, respectively [7]. Among noncommunicable diseases, the contribution of air pollution to the incidence of lung cancer is 26%, and its contributions to cardiovascular disease and to stroke are 17% and 12%, respectively [8,9].

Pollution-related diseases have soared since the rapid development of urbanization and industrialization in China. This has affected healthcare services profoundly. Outpatient and inpatient services are the main ways to access healthcare [10]. According to the China Health Statistics Yearbook, it is estimated that compared with 2010, in 2020, the number of outpatient visits will rise by 11.4 billion, and the number of inpatient visits will increase by 279 million, with average annual growth rates of 6.9% and 7.0%, respectively. Among all healthcare services, treatment for pollution-related diseases such as cardiovascular diseases, respiratory diseases, digestive tract and intestinal diseases, and cancer generate serious disease burdens in China.

Previous researchers have conducted various studies to explore the relationship between environmental pollution and health or healthcare services in China. The relationship with air, water, and solid pollution has been discussed extensively. Song conducted research to estimate the health burden attributable to ambient PM2.5 across China by using the exposure-response model [11]. Zeng applied spatial autocorrelation analysis, hot spot analysis, and spatial empirical analysis to explore the spatial distribution of PM2.5 and described its relationship with healthcare services [12]. Wang revealed that water pollution was negatively associated with health outcomes by using a random-effects model, a random-effects logit model, and a mediator model [13]. Yang explored and assessed the impact of heavy metal soil pollution induced by industrial and agricultural activities in China [14]. Pollution can cause several diseases, such as respiratory diseases [15,16], cardiovascular diseases [9,17], gastrointestinal diseases [18,19], reproductive diseases [20,21], and cancer [22,23]. Pollution in China also changed the healthcare situation by increasing the disease burden. For example, Yang estimated the effect of air pollution exposure on household healthcare expenditure by using the China Urban Household Survey (UHS) Database [24]. Taj researched the relationship between primary healthcare (PHC) visits, inpatient admissions, emergency room visits, and air pollution concentrations [25]. Zhe and Hong analyzed the relationship between air pollution and outpatient visits [26,27].

Although there have been comprehensive and systematic studies about environmental pollution and health or healthcare in previous studies, some research fields can still be explored. On the one hand, the spatial properties of pollution have still not been widely investigated. Air pollution could spread over adjacent provinces with no regard for political boundaries [28]; water pollution upstream could cause destructive effects on downstream regions [29]; solid waste can contaminate soil without being limited by space [30]. To an extent, some studies have analyzed the spill-over effects of air pollution [12]; however, these spill-over effects have still not been fully considered, such as spill-overs due to water pollution and solid-waste pollution. Apart from that, specifically, there has been no detailed discussion about the relationship between outpatient services, inpatient services, and environmental pollution. Healthcare services aim to address different kinds of diseases, disease severities, and groups of people. Therefore, the main contributions of this paper are listed as follows:

(1) This study aims to explore the spatial characteristics and distributions of different pollutants and of the utilization of healthcare services in China to provide proper guidance on spatial policies for the government.
(2) Spatial econometric models were applied in this study to assess the spill-over effects of different pollutants on healthcare service utilization, that is, to identify the relationship between pollution in the local region and healthcare service utilization in geographically or economically adjacent provinces.
(3) Inpatient and outpatient services are two different services used by different groups of people. In this study, we explored the direction and magnitude of the effects of environmental pollution on inpatient and outpatient services.

## 2. Materials and Methods

### 2.1. Data Source

This study applied province-level data on outpatient services, inpatient services, environmental pollution, and other socioeconomic characteristics from 2006–2017 in China. Table S1 shows detailed information on all analyzed data. The outpatient and inpatient data were obtained from the China Health Statistics Yearbook (CHSY) (http://www.nhc.gov.cn/zwgk/tjnj1/ejlist_3.shtml (accessed on 11 February 2021)) and China Health and Family Planning Statistical Yearbook (CHFPSY) (http://www.nhc.gov.cn/zwgkzt/tjnj/list.shtml (accessed on 11 February 2021)), issued and published by the National Health Commission of China, while the environmental pollution and socioeconomic data were obtained from the China Statistical Yearbook (CSY) (http://www.stats.gov.cn/enGliSH/Statisticaldata/AnnualData/ (accessed on 11 February 2021)) published by the National Bureau of Statistics of China. Table 1 shows detailed information on the variables and data sources.

### 2.2. Variables

#### 2.2.1. Dependent Variables: Outpatient and Inpatient Care Services

Outpatient and inpatient care services reflect health status to an extent. Outpatient care services normally admit patients with mild symptoms. Doctors can make a preliminary diagnosis after a series of auxiliary examinations or medical judgements. A regular exam with a primary care physician and an appointment with a psychologist are both examples of outpatient care. In addition, emergent cases are also regarded as outpatient care [31,32,33]. There is a distinct difference between inpatient and outpatient care: an inpatient is someone admitted to the hospital to stay overnight. Naturally, the diseases experienced by these patients are serious and sometimes fatal. In summary, outpatient and inpatient services are core indicators of the health burden and for medical services. In this study, outpatient visits and the number of hospitalizations were chosen to represent healthcare services as the dependent variables.

#### 2.2.2. Independent Variables: Environmental Pollution

Environmental pollution can affect people’s health. There are many kinds of pollution, while only air pollution, water pollution, solid waste, and domestic garbage were specifically included in this study. The main air pollutants are smoke and dust, particulate matter (PM2.5), sulphur dioxide (SO_2_), and nitrogen dioxide (NO_2_) [34,35]. Water pollution includes chemical oxygen demand (COD), ammonia nitrogen, nitrogen, phosphorus, petroleum, volatile phenol, plumbum, mercury, hexavalent chromium, chromium, and arsenic [36]. There are different types of solid wastes, such as metallurgical solid waste residue [37]. In this study, the industrial and residential water discharge amounts were chosen to capture water pollution; air pollution is represented by the amount of gas emissions from industry and car exhaust. PM2.5 is used as an independent variable due to its importance; solid wastes are denoted by the amount of discharge from industrial pollutant emissions and household waste disposal.

#### 2.2.3. Control Variables

According to the determinants of health summarized by the World Health Organization (WHO), the social and economic environment, the physical environment, individual characteristics, and individual behaviors are four main factors influencing health. Outpatient visits and inpatient visits are affected not only by environmental factors but also by many other determinants of health. In line with previous studies, this study included the following control variables: per capita gross domestic product, the ratio of the urban population to the total population, the ratio of the population aged 0–14 and the ratio of the population aged above 65 to the total population, and the ratio of the population with an associate’s degree or higher.

The variable measurements, codes, and descriptions are shown in Table 2.

### 2.3. Methods

#### 2.3.1. Spatial Autocorrelation Test

Moran’s I was proposed by Australian statistician Patrick Alfred Pierce Moran in 1950 [38]. It is regarded as the best method for testing for spatial autocorrelation. There are two different types of Moran’s I that each have different functions. The global Moran’s I is used to determine whether the variables are spatially clustered, while the purpose of the local Moran’s I is to identify the specific clusters into which those variables are grouped. There are four types of cluster: high-high (HH) clusters, low-low (LL) clusters, high-low (HL) clusters, and low-high (LH) clusters. The identification of clusters is based on the coefficients on the indicators [39,40,41,42,43]. The specific calculation formulas are as follows:

(1) $${\mathrm{Global}\;\mathrm{Moran}}^{’}s\;I=\frac{n\sum_{i=1}^{n}\sum_{j=1}^{n}w_{ij}\left(x_{i}-\bar{x}\right)\left(x_{j}-\bar{x}\right)}{\sum_{i=1}^{n}\sum_{j=1}^{n}w_{ij}{\left(x_{i}-\bar{x}\right)}^{2}}$$

(2) $${\mathrm{Local}\;\mathrm{Moran}}^{’}s\;I=\frac{\left(x_{i}-\bar{x}\right)}{m_{0}}\sum_{j}W_{ij}\left(x_{j}-\bar{x}\right);\;m_{0}=\sum_{i}{\left(x_{i}-\bar{x}\right)}^{2}/n$$

where *n* represents the number of observations, $x_{i}$ denotes the total outpatient and inpatient visits per capita in province *i*, $x_{j}$ is the total outpatient and inpatient visits per capita in province j, $\bar{x}\;$indicates the mean value of the variable *x*, and $w_{ij}$ symbolizes the spatial weight matrix for *i* relative to *j*. In this study, the spatial weight matrix aggregates 31 geographical units and captures the spatial effects between the adjacent provinces *i* and *j*. Whether there are spatial influences depends on the geographical and economic relationship between the provinces. Therefore, the value of $w_{ij}$ is determined by the boundaries between provinces, the distances between different provinces, and the economic relationships between the provinces.

The range of Moran’s I is [–1,1]. If the value of the index is approximately −1, there is no spatial similarity between adjacent provinces. Specifically, this indicates that a province with a high number of outpatient visits or a high level of pollution borders other provinces with a low number of those visits or a low level of pollution. When the value is near 1, there are spatial similarities, namely, a region with a high number of outpatient visits or a high level of pollution is adjacent to another region with similarly high values for those variables. When the value is approximately 0, there is no spatial autocorrelation in the research area, and all the variables are randomly distributed among the provinces. In this study, the Moran’s I for pollution in China from 2006–2017 is calculated.

In regard to spatial autocorrelation, spatial matrices should be introduced into the analysis to describe the relationships between the provinces. There are three kinds of matrices used in this study, namely, the spatial contiguity matrix M_1_, the spatial distance matrix M_2_, and the spatial economic matrix M_3_ [44,45].

**Figure 1:**
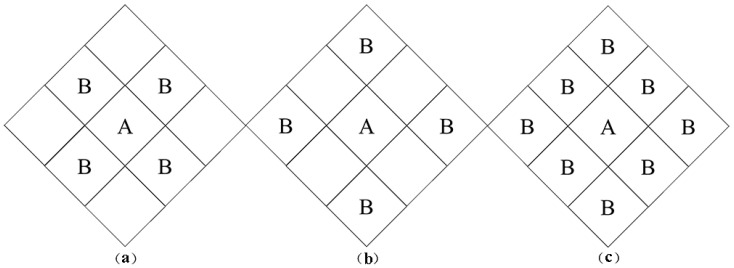
Different types of spatial contiguity matrices. (**a**) Rook adjacency; (**b**) Bishop adjacency; (**c**) Queen adjacency.

In deriving the spatial contiguity matrix, the spatial weight matrix is mainly determined according to adjacency relationships. When region i is adjacent to region j, W_ij_ = 1; otherwise, W_ij_ = 0. For the lattice of spatial adjacency relationships, the adjacent provinces can have a common boundary or a common vertex, and the Rook matrix, Bishop matrix, and Queen matrix can be defined accordingly [46,47], as shown in Figure 1.

In Figure 1a, the units adjacent to A are those units B that have a common boundary with A, called Rook adjacency; in Figure 1b, the units adjacent to A are those units B that have a common vertex with A, called Bishop adjacency; in Figure 1c, the units adjacent to A are those units B that have a common boundary or a common vertex, and this is called Queen adjacency. Queen adjacency is the superposition of Rook adjacency and Bishop adjacency.

In addition to the adjacency relationships that can describe the relationships between spatial units, distance is also an important indicator of spatial patterns. Tobler’s first law of geography tells us that everything is related to other things, but similar things are more closely related. Therefore, the shorter the distance between two provinces is, the closer their relationship and the greater the weight W_ij_. However, in spatial econometrics, distance is not only a narrowly defined distance but also a generalized distance. The narrowly defined distance usually refers to the physical distance, which is only measured from a geographical perspective, for example, the distance between the centroids of two regions or between the administrative centers. The generalized distance includes various forms of virtual distances, such as economic distance, social distance, or temporal distance. The reason for investigating this kind of virtual distance is that in spatial econometrics, we often pay attention to the economic significance of variables and their regional relevance in economic development and the social culture. This level of significance goes beyond the simple geographic distance. In many cases, two regions with very close physical distances do not necessarily produce an agglomeration of a certain economic phenomenon. For example, Anhui Province is adjacent to Zhejiang Province and Jiangsu Province, but the strength of economic development in Jiangsu Province and Zhejiang Province is obvious. Their relationships with Anhui Province are weaker, so we have reason to believe that the “economic distance” between Jiangsu Province and Zhejiang Province is shorter, and the “economic distance” between Anhui Province and Jiangsu and Zhejiang is farther. The spatial weight coefficient of the former is compared with that of the latter. The larger one is, the stronger the spatial correlation. Based on this idea, this study includes each of these three matrices.

#### 2.3.2. Spatial Econometric Model

This study applied a spatial econometric model to explore the empirical relationship between healthcare services and environmental pollution in China using yearly panel data from 2006 to 2017. The direct and spill-over effects were revealed in this study. Three widely used spatial econometric models were introduced to identify all effects: the spatial lag panel model (SLPM) (Formula (3)), the spatial error panel model (SEPM) (Formula (4)), and the spatial Durbin panel model (SDPM) (Formula (5)). The models can be expressed as [48]:

(3) $$y_{it}=\rho W_{ij}y_{it}+X_{it}^{\prime }\beta +\alpha +\mu_{i}+\gamma_{t}+\varepsilon_{it}\;i=1,\;2,\;3\ldots 31\;t=2006,\;2007\ldots 2017$$

(4) $$y_{it}=X_{it}^{\prime }\beta +\alpha +\mu_{i}+\gamma_{t}+\varepsilon_{it\;}\varepsilon_{it}=\lambda \sum_{j=i}^{n}W_{ij}\varepsilon_{it}+\tau_{it}\;i=1,\;2,\;3\ldots 31\;t=2006,\;2007\ldots 2017$$

(5) $$y_{it}=\rho W_{ij}y_{it}+X_{it}^{\prime }\beta +W_{ij}y_{it}\theta +\alpha +\mu_{i}+\gamma_{t}+\varepsilon_{it}\;i=1,\;2,\;3\ldots 31\;t=2006,\;2007\ldots 2017$$

In the SLPM equation above, the regression coefficient is represented by β. Similar to the definition in a normal ordinary least squares regression analysis, it reveals the effects of environmental pollution on healthcare services. $W_{ij}\;$is the spatially weighted 31 × 31 matrix for the provinces of China. The estimation of coefficient ρ provides the slope of the function, which reflects the level of influence of surrounding provinces on the distribution of healthcare services; $\mu_{i}\;$and $\gamma_{t}$ represent spatial fixed effects and time fixed effects, respectively. Apart from the existing parameters in the SLPM, in the formula for the SEPM, the spatial dependence of all the variables was measured by the parameter λ, which represents the magnitude and direction of the effect of one variable in a surrounding region on the same variable in a given region. *ε* denotes the spatial autoregressive error term. In the SDPM formula, *θ* represents the influence of the dependent variables in adjacent areas on the independent variables in a given area.

The spatial lag model indicates that the spatial correlations occur over time, which means that the spatial weight matrix appears to be related to the previous variables. It mainly explores whether each variable has a spill-over effect in a region, that is, whether the variables of specific provinces are affected by those in their adjacent areas. The spatial error model indicates that the spatial effect is in the error term, that is, the spatial weight matrix is placed in an unobservable error term, which reflects that the dependent variables of one specific province are associated with the independent variables of that province as well as the error term. The SDPM model is used to address the circumstance where the dependent variables could be explained by the independent variables of both a specific area and its surrounding provinces.

### 2.4. Software

The spatial weight matrix was generated using GeoDa (Version 1.8.61, the University of Chicago, and Chicago, IL, USA), and STATA 15.0 (Version 15.0, StataCorp., College Station, TX, USA) was employed to estimate the spatial panel models.

## 3. Spatial Distribution and Spatial Autocorrelation Analysis

### 3.1. Descriptive Analysis

Table 3 presents the descriptive statistics for the sampled Chinese provinces. Let us take OV as an example: the average number of outpatient visits per capita was 7.76 visits with a standard deviation of 11.68. Compared with IV, OV showed a high degree of dispersion. The maximum value of OV was more than 500 times larger than its lowest value, indicating a distinct difference in access to healthcare across China. In summary, detailed information on the two dependent variables, five independent variables, and six control variables is shown.

**Figure 2:**
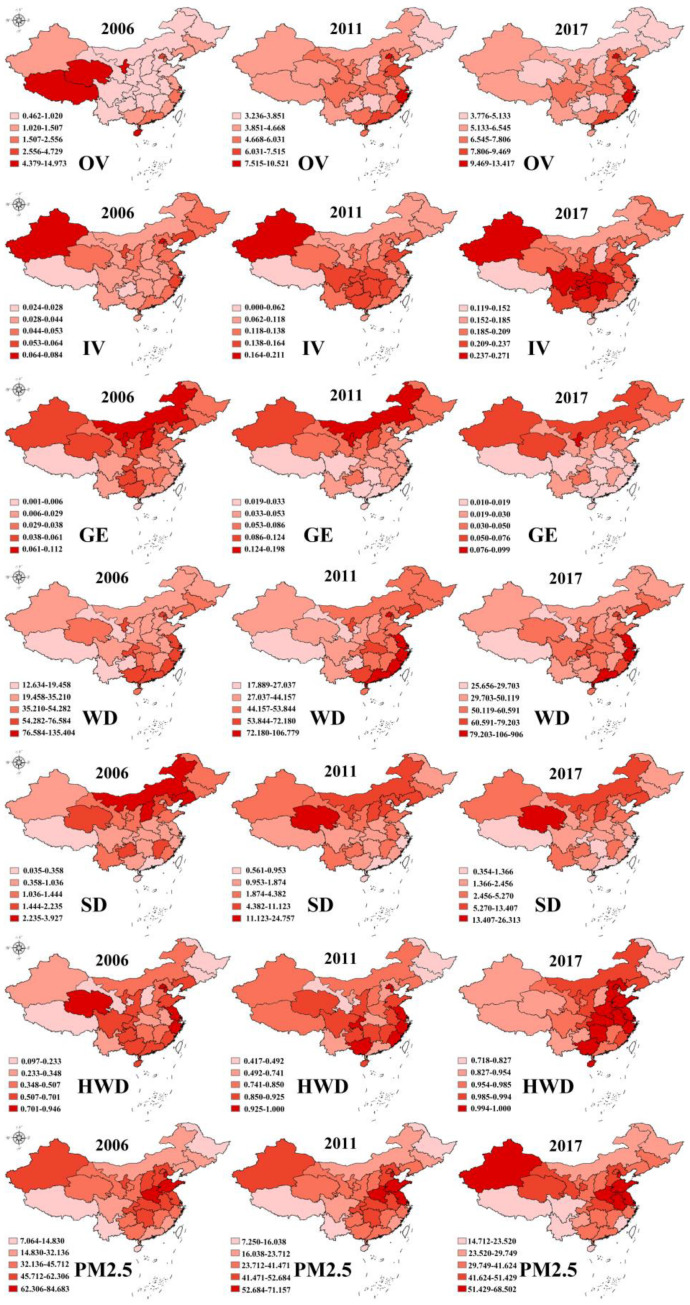
Hierarchical maps of the densities in health resources ^1^. ^1^ OV: outpatient visits per capita; IV: inpatient visits per capital; ED: effluent discharge; GE: gas emission; SD: solid discharge; PM2.5: average PM2.5 concentration; HWD: household waste disposal.

Figure 2 displays the spatial distribution of the main dependent and independent variables: outpatient visits, number of hospitalizations, effluent discharge, waste gas emissions, solid-waste discharge, household waste disposal, and average PM2.5 concentration.

A longitudinal (temporal) comparison of the data from 2006 with that from 2017 shows that there was a slight increase in outpatient visits and hospitalizations by 2017 in various provinces. From the perspective of horizontal (spatial) comparisons, in 2006, western areas such as Xizang, Qinghai, Xinjiang, and Ningxia had the highest number of outpatient visits per capita, indicating that outpatient services were satisfying the needs of patients. Over time, several medical resources flowed to more developed regions such as Shanghai, Beijing, and Zhejiang. The number of hospitalizations indicate a different situation: there was almost no change throughout the whole research period. Specifically, inpatient services in Xizang were insufficient compared with those in other provinces, while Xinjiang, Hubei, Hunan, Sichuan, Chongqing, and Guizhou had the highest number of hospitalizations from 2006 to 2017.

Regarding the independent variables, the air pollution results highlighted an interesting difference between the northern and southern regions. High pollution areas were mainly concentrated in northern provinces such as Neimenggu, Shanxi, and Ningxia, while low pollution regions were distributed in southern provinces such as Hainan and Guangdong. Regarding the PM2.5 concentration, the same situation was found, but in the north, the highest levels were discovered in the Huabei Plain (Hebei, Shandong, Henan, Jiangsu, and Anhui). This distribution is related to provincial economic structures and meteorological factors. For example, Neimenggu and Shanxi are famous for their coal industry in China, while Hainan and Guangdong are well known for tourism. From a meteorological perspective, northern China is drier than the southern region.

The situation with water pollution was relatively different from that with air pollution. The regions with heavy pollution were mainly distributed along the south-eastern coastal provinces. Shanghai, Jiangsu, Fujian, Guangdong, and Zhejiang were found to be high-pollution regions. In coastal regions, it is easier to discharging sewage into the ocean. Apart from that, the southeastern provinces are the richest places in China. Regarding solid waste, the highest amount of solid discharge was found in some of the northern regions. This could be attributed to provincial economic structures. Household waste disposal is associated with economic development levels. Developed region were found to have a high rate of waste disposal.

### 3.2. Spatial Autocorrelation Analysis

The global Moran’s I for effluent discharge, waste gas emissions, solid discharge, PM2.5 concentrations, and household waste disposal are shown in Table 4

This indicates that almost all the coefficients on the environmental variables are positive and reach the 5% significance level. Specifically, the distribution of all variables could be described as clusters of high values or clusters of low values. Spatial correlations should be considered when analyzing the relationship between healthcare services and environmental pollution. (M_1_)

**Figure 3:**
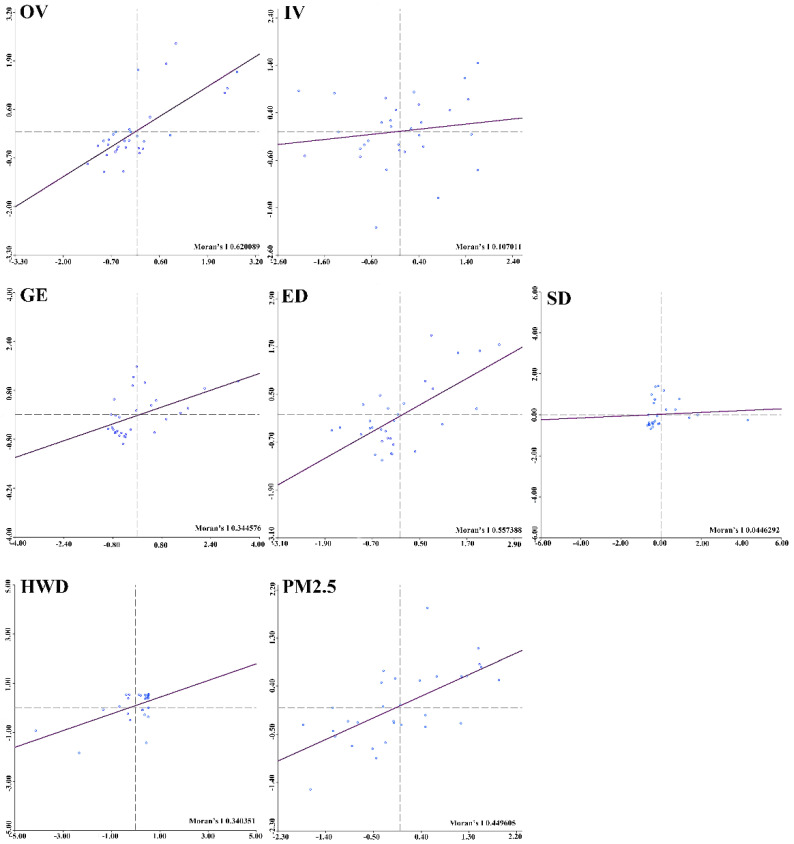
Local Moran’s I scatter plots ^1^. ^1^ OV: outpatient visits per capita; IV: inpatient visits per capital; ED: effluent discharge; GE: gas emission; SD: solid discharge; PM2.5: average PM2.5 concentration; HWD: household waste disposal.

To describe the local agglomeration characteristics among all provinces in China, scatter plots were drawn and hot spot analyses were conducted. Figure 3 displays the scatter plots of the local Moran’s I for all main independent and dependent variables using matrix M_1_ for all provinces from 2006 to 2017. Specifically, the two coordinate axes represent the value of variable in a certain province and in its adjacent regions. The scatter plot was divided into four quadrants. Based on quadrant theory, the first quadrant represents high-high (HH) clusters, which means that the specific region and its surrounding area both had high values for certain variables. Next, the second quadrant represents low-high (LH) clusters, in which the provinces with a low observed value are surrounded by regions with high values. The third quadrant represents low-low (LL) clusters, and the fourth quadrant represents high-low (HL) clusters, which are analogous to the LH clusters.

As shown in Figure 3, from 2006 to 2017, the majority of the provinces were located in the HH and LL clusters, revealing that most regions were positively spatially correlated. This means that the distributions of healthcare services and pollution in China from 2006 to 2017 were not random but exhibited positive spatial autocorrelation. Taking OV as an example, seven provinces were located in HH cluster areas, 16 provinces were found in the LL clusters, and only eight provinces were discovered in the LH or HL cluster regions.

**Figure 4:**
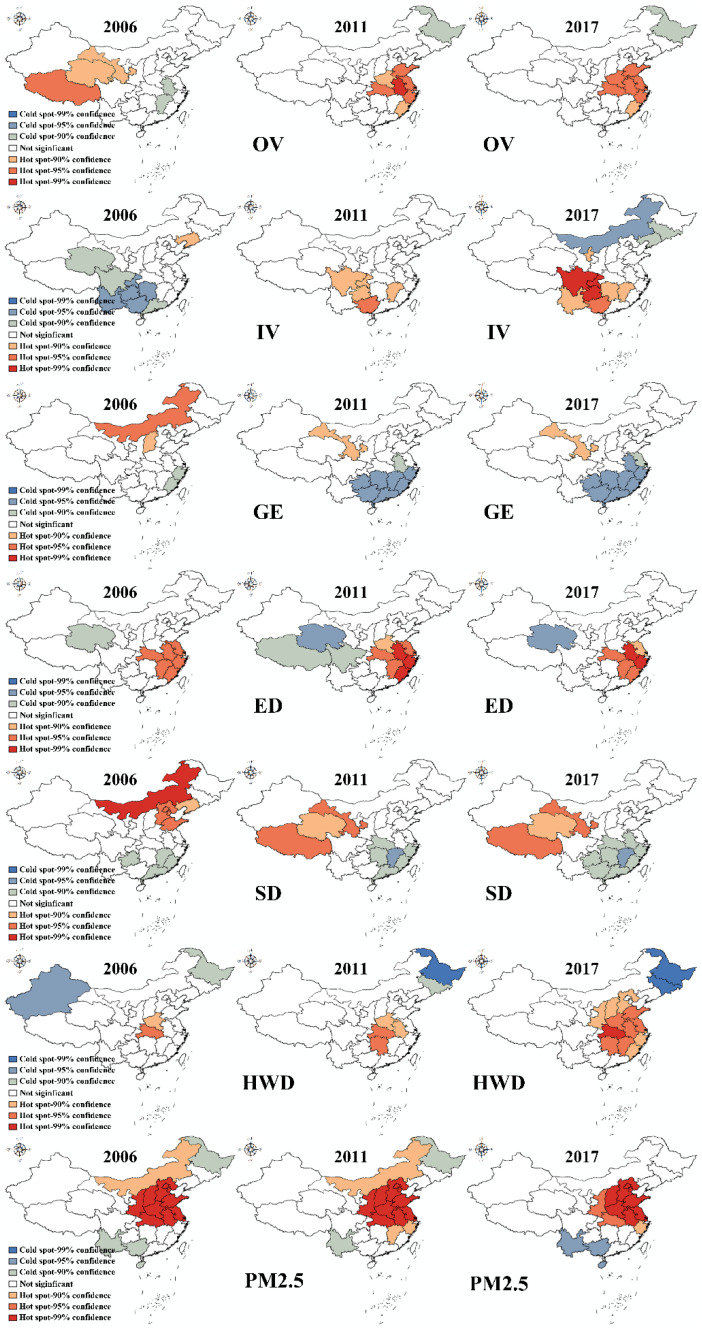
Hot spot analyses ^1^. ^1^ OV: outpatient visits per capita; IV: inpatient visits per capital; ED: effluent discharge; GE: gas emission; SD: solid discharge; PM2.5: average PM2.5 concentration; HWD: household waste disposal.

Figure 4 displays the results for all independent and dependent variables for all provinces from 2006 to 2017 from the hot spot analysis (namely, the Getis-Ord Gi* method). This tool is meant to identify statistically significant hot spot or cold spot spatial clusters. The z-score, *p*-value, and confidence level are the main explanatory parameters. The z-scores and *p*-values reveal statistical significance. A high z-score and a small *p*-value for a feature indicate spatial clustering of high values. A low negative z-score and a small *p*-value indicate spatial clustering of low values. The higher (or lower) the z-score is, the more intense the clustering. A z-score near zero indicates no apparent spatial clustering. In this study, all *p*-values representing different significance levels are displayed. As shown in Figure 4, for OV in 2006, cold spots were found in the southeastern region, namely, in Anhui and Jiangxi Provinces, while hot spots were discovered in provinces in western China, including Xizang, Qinghai, and Gansu. In 2011, there were some changes in the distribution of cold and hot spots. Specifically, Heilongjiang Province developed into a cold spot. In contrast, a cluster of hot spots was found in eastern China, containing Shandong, Henan, Hunan, Anhui, Jiangsu, Zhejiang, Fujian, and Shanghai. There is a dramatic change from 2006 to 2017, but there was little change in 2017 relative to 2011. The situation for the other variables can be seen in Figure 4.

## 4. Empirical Analysis and Discussion

After a series of tests for the selection of a spatial panel model, a SPDM was used in our study. The Moran’s I and LM tests were applied to examine the level of spatial autocorrelation, followed by the Wald test and LR test, which can identify the most appropriate model among the SLPM, SEPM, or SPDM. After that, the Hausman test was used to determine the specific kind of SPDM to use, namely, a fixed-effects model or a random-effects model. If a fixed-effects model is chosen, then the last step is to decide among individual fixed effects, spatial fixed effects or both. Therefore, on the basis of the Hausman test, the SPDM with random effects was chosen to analyze the correlation between pollution and healthcare.

### 4.1. Empirical Results of Spatial Panel Models

The empirical results of the spatial panel model based on three different spatial weight matrices are presented in Table 5. Columns (1), (2), and (3) show the results of different pollutants and control variables on outpatient services based on the spatial contiguity matrix M_1_, spatial distance matrix M_2,_ and spatial economic matrix M_3,_ respectively, while columns (4), (5), and (6) show the findings for the effects of all independent variables on inpatient services.

The coefficients on gas emissions for outpatient visits based on the spatial contiguity matrix M_1_ and spatial economic matrix M_3_ were positively related to outpatient visits, which indicates that an increase in air pollution leads to an increase in outpatient visits. The reason behind this is that air pollution could place people at serious risk of cardiovascular and respiratory disease. Therefore, outpatient visits soared in contaminated regions. However, there was no relationship between PM2.5 and outpatient visits, which is different from the findings of previous research [49,50]. The possible reason for this may be related to the roughness of the monthly PM2.5 data at the provincial level. In summary, air pollution influenced the usage of outpatient services.

In consideration of the influence of effluent discharge on outpatient visits, all coefficients were significantly positive at the 5% level. This shows that water pollution could contribute to the growth in outpatient visits. Additionally, when the spatial distance matrix was used in the analysis, the coefficients on effluent discharge were significant. Comparing this finding with the results from using M_1_ and M_2_ indicates that outpatient visits were not only affected by water pollution in the local region but also by the pollution in nearby regions, while there was no impact on outpatient visits from variables in adjacent regions or in economically related regions.

When estimated using the spatial contiguity matrix M_1_ and spatial distance matrix M_2_, solid waste was found to be positively associated with outpatient visits. To an extent, this suggests that an increase in solid waste in geographically close regions or adjacent areas causes an increase in outpatient visits. However, in regard to household waste disposal, all relevant coefficients were positive regardless of which matrix was used. The interpretation for this is that high levels of household waste disposal abnormally increase outpatient visits, which is different from the findings of other studies [51]. A possible reason for this is that rich regions with high numbers of outpatient visits tend to be capable of recycling solid waste. This leads to a spurious correlation between household waste disposal and outpatient visits.

Last, for the control variables, almost all demographic variables were positively related to outpatient visits. The higher the total population and the higher the share of the urban population, population aged 0–14, and population aged above 65, the higher the number of outpatient visits. Urban citizens are able to access healthcare services more easily. In addition, children and elderly individuals are more susceptible to diseases. Interestingly, outpatient visits in some regions were negatively associated with those of geographically and economically adjacent provinces. The reason behind this is that the healthcare services in a given region not only satisfy the basic needs of local people but also attract patients from other places. Part of the population in some regions may be more easily attracted to other regions when local healthcare services cannot solve their issues. This phenomenon is quite common in western China. Regarding the economic variables, GDP per capita is positively associated with outpatient visits. Rich regions tend to have developed healthcare services with advanced technology and a reasonable amount of health resources. The majority of the population would be drawn to richer areas to receive better services. There were no significant results concerning the education variable.

From the perspective of inpatient visits, almost all coefficients related to pollution were insignificant apart from that on effluent discharge when using the spatial contiguity matrix M_1_. There was a distinct difference between outpatient and inpatient services. This situation can be explained by the difference between long-term and short-term effects. Outpatient services normally are related to diseases with mild symptoms and that can be more easily affected by environmental pollution. Therefore, the relationships between outpatient services and the environmental variables were more evident than those between inpatient services and the environmental variables. In contrast, patients with serious diseases must accept standard treatments in inpatient institutes. There was little evidence of a distinct linkage between environmental factors and inpatient services. In addition, concerning spatial relationships, solid waste was positively related to inpatient services, which means that solid pollution in surrounding regions could lead to an increase in inpatient visits in specific provinces, while solid pollution from geographically close regions and economically close regions was not related to outpatient visits.

Considering the other control variables, the demographic variables were positively associated with inpatient visits, similar to their relationship with outpatient visits; naturally, the larger the population and the greater the share of the urban population, population aged 0–14, and population aged above 65, the more inpatient visits there were. At the same time, other places with high levels of inpatient services could be attracting more people from other areas.

The spill-over and direct effects estimated by the above spatial economic models cannot be interpreted directly. The spill-over and direct effects of the environmental variables and the control variables were determined, and the results are given in Table 6 and Table 7.

### 4.2. Decomposition of Direct and Spill-Over Effects

Table 5 reports the direct effects of the independent variables on the different health resources. For the effect of gas emissions on outpatient services, the coefficient based on the economic matrix was significant at the 1% level, which suggests that for every 1% increase in gas emissions, outpatient visits grow by 0.350%. All coefficients on effluent discharge based on the three matrices were significant (0.583, 0.738, and 0.363). These can be interpreted as follows: for every 1% increase in effluent discharge, outpatient visits increase by 0.583%, 0.738%, and 0.363%, respectively. For solid waste, outpatient visits increase by 0.127% and 0.161% when solid waste discharge increases by 1%.

The direct effects of the control variables showed a positive relation between outpatient visits and other socioeconomic factors when using all three matrices. Let us take the coefficient of spatial contiguity matrix M_1_ as an example: when the total population increases by 1%, outpatient visits grow by 0.595%. Similarly, outpatient visits grow by 1.560% after a 1% increase in the urban population share. Finally, 1% growth in the percentage of the population aged 0–14 increases outpatient visits by 3.858%.

There was a distinct difference in the results for inpatient visits compared with those for outpatient services. Gas emissions made no difference in the number of inpatient services provided. Only the coefficients on effluence discharge using M_1_ and on solid waste using M_3_ are significant, which suggests that with every 1% increase in effluence discharge and solid waste, inpatient visits increase by 0.151% and 0.048%, respectively. At the same time, the total population, urban population, and percentage of the population above 65 could also contribute to the growth in inpatient visits. However, there was one interesting finding: the percentage of the population above 65 was closely related to inpatient services, while the percentage of the population that is young was related to outpatient visits. This may be caused by different preferences for healthcare services among people of different age groups.

Table 6 reports the spill-over effects of the independent variables on different healthcare services in surrounding areas. As was the case for outpatient visits per capita and inpatient visits per capita, the environmental factors had almost no impact on outpatient services in the surrounding regions. In consideration of the demographic factors, the total population, urban population, and population aged above 65 had spill-over effects on outpatient visits in other areas. A 1% increase in the total population in the local region was related to decreases of 0.663% and 0.767% in adjacent regions and economically close regions, respectively. In regard to the urban population, a 1% increase in certain provinces corresponded to a 22.985% drop in inpatient visits. For the population above 65, the coefficient is −9.276. Concerning the economic factors in the model, a 1% increase in GDP per capita was related to an increase in inpatient visits (according to the three matrices, the increase was 1.539%, 4.654%, and 4.179%, respectively.)

From the perspective of inpatient visits, it was estimated that with a 1% increase in solid waste discharge in one specific province, the inpatient visits per capita in adjacent counties would increase by 0.559%. Concerning the control variables, the total population, urban population, and population aged 0–14 were all associated with increases in inpatient visits in adjacent areas. Considering the coefficient estimated using the spatial contiguity matrix M_1_ for instance, a 1% increase in the total population in the local province was associated with a 0.801% decrease in inpatient visits in the adjacent region, a 1% increase in the urban population in the local region leads to a 4.592% decrease in inpatient visits in the surrounding regions, and a 1% increase in the percentage of the population aged 0–14 contributes to a 6.135% decrease in inpatient visits in the surrounding regions. In regard to the economic determinants, the coefficients estimated using the spatial contiguity matrix M_1_ and the spatial economic matrix M_3_ were positive. This suggests that a 1% increase in GDP per capita in the local region is associated with a 1.002% increase and 1.085% increase in inpatient visits in the surrounding provinces or in economically related regions. Interestingly, educational levels also play a role in the utilization of healthcare services, and other studies have found similar results for specific diseases [52].

## 5. Conclusions

Using panel data on all provinces in China from 2006 to 2017, this research used spatial autocorrelation analysis and a SDPM with random effects to explore the basic spatial characteristics of healthcare services and their spatial relationships with environmental variables based on three spatial weight matrices. The main findings of this study were as follows:

(1) The utilization of healthcare services and environmental pollution were not randomly distributed in China. There were distinct spatial cluster characteristics according to the global Moran’s I analysis. Healthcare services were affected not only by environmental factors but also by the pollution of geographically and economically related regions.
(2) Environmental pollution could pose a threat to the health of humans. Regardless of whether the pollution is from the local region or another related province, the empirical analysis indicates that the growth of pollutants could contribute to the increase in outpatient and inpatient visits. For example, with every 1% increase in gas emissions in the local region, outpatient visits grow 0.350% in that specific region.
(3) Environmental pollution exhibited spill-over effects on healthcare services. Local pollution can cause an increase in healthcare services in the same region, while pollution in other geographically or economically related regions could also affect healthcare services in the local region. Naturally, some forms of pollution, such as air pollution, can spread towards other places. Effluent discharge from upstream flows downstream. Economically related regions normally develop industries at the same pace, which may lead to similar contamination situations.
(4) Specifically, pollution showed different degrees of influence on healthcare services, which means that the impact of environmental pollution on the number of outpatient visits is greater than its impact on that of inpatient visits. Almost all relationships between pollution and outpatient visits were significant, while there was no significant relationship between pollution and inpatient visits. Normally, outpatient services are for emergencies or diseases with mild symptoms, while inpatient services are aimed at diseases with serious symptoms or those requiring surgery. Although environmental pollution could cause diseases with either mild or severe symptoms, it is difficult to distinguish these factors from other determinants of disease.
(5) This finding was related to the control variables. Different age groups were associated with different healthcare services. Children aged 0–14 tended to receive outpatient services, while elderly people were more likely to receive inpatient services. This may be explained by the disease spectrum across different age groups. In addition, demographic factors showed distinct spill-over effects, and the high need for healthcare services in some places could lead to a decrease in the provision of healthcare services in geographically and economically close regions.
(6) The lasting finding was associated with economic development, in some extent, the relationship between economic growth and health with environmental pollution as mediator variable was still unclear. In this study, per capita GDP was included in as independent variable, and the positive association was found towards this variable. The reason behind this was the attraction of rich regions which owned better medical services. However, economic development may cause environmental pollution and changes of healthcare services. This study was unable to state that relationship. There were two main opinions about this relationship in research world: “economic growth-heavy environmental pollution-bad health-heavy healthcare needs”, “economic growth- transformation of industries-better environment-less healthcare needs”. The first change improves healthcare needs in local regions, while the other changes that in other regions. To sum up, the relationship between economic growth and health with environmental pollution as mediator variable was complicated and this study failed to prove it.

According to the conclusions above, some policies can be proposed:

(1) At the governmental level, authorities from all levels should not only participate in the formulation and implementation of basic environmental protection policies but also enhance their cooperation with other authorities. On the one hand, the management of health and environmental protection belong to different administrative divisions in China, so authorities in all divisions should work together to address this problem. On the other hand, environmental pollution showed spill-over effects, so regional and national cooperation should be considered.
(2) From the perspective of a company or industrial organization that may cause pollution, apart from obeying the basic requirements issued by the government, the relevant companies should pay more attention to the location of their spill-over effects. Most importantly, the location of factories should be distant from residential areas and the community. In addition, all factories should obtain information on their pollutants and the damage caused thereby, as well as on the areas affected by that damage. This could help effective and fast measures be implemented when an emergency occurs.
(3) From the perspective of communities and citizens, it is vital to learn and understand the damage caused by environmental pollution and to take measures towards alleviating this problem. Specifically, it would be better for communities and citizens to have access to information about surrounding factories and their possible pollutants in order to take necessary precautions against those pollutants. In addition, citizens who live in unpolluted areas should try to avoid staying in places with heavy pollution for a long time, such as mining areas, waste incineration areas, and regions with contaminated water.

Limitations and strengths:

(1) Limitations

To start with, this study failed to examine the relationships between other variables and healthcare services with environmental pollution as mediator variable. These associations were widely discussed in the academic world. Besides, the relationship between health resources such as health workforce, bed numbers, equipment, and environmental pollution was not researched.

(2) Strengths

To our knowledge, this was the first study using the effluent discharge, waste gas emission, solid discharge, and household waste disposal as environmental pollution variables in discussion regarding its relationship with healthcare services. Second, this study explored the change of environmental pollution and healthcare services in China from 2006 to 2017.

## Figures and Tables

**Table 1:** Variables and data sources.

Variables	Research Subjects	Years	Data Resources
Outpatient visits	31 provinces	2006–2017	CHSY, CHFPSY
Inpatient visits	31 provinces	2006–2017	CHSY, CHFPSY
Water pollution	31 provinces	2006–2017	CSY
Air pollution	31 provinces	2006–2017	CSY
solid wastes	31 provinces	2006–2017	CSY
PM2.5 concentrations	31 provinces	2006–2017	CSY
Domestic waste disposal	31 provinces	2006–2017	CSY
Per capita GDP	31 provinces	2006–2017	CSY
Urban population	31 provinces	2006–2017	CSY
Population aged 0–14	31 provinces	2006–2017	CSY
Population aged above 65	31 provinces	2006–2017	CSY
Education level	31 provinces	2006–2017	CSY

**Table 2:** Variables and data sources.

Variable Type	Variable Name	Measurement	Code	Description
Dependent variable	Medical care	Outpatient visits per capita	OV	Number of the outpatient visits divided by the population
		Inpatient visits per capital	IV	Number of the inpatient visits divided by the population
Independent variables	Environmental pollution	Effluent discharge	ED	Amount of effluent discharge divided by the population
		Waste gas emission	GE	Amount of gas emission divided by the population
		Solid discharge	SD	Amount of solid discharge divided by the population
		Average PM2.5 concentration	PM2.5	The average values of PM2.5 concentrations in the form of the natural logarithm
		Household waste disposal (%)	HWD	The ratio of household waste disposal
Control variables	Socioeconomic conditions	Gross domestic product per capita	GDP	Real gross domestic product divided by the population
	Demographic conditions	Total population	TP	The total population of all counties
		The proportion of the urban population (%)	PUP	A total population divided by urban population
		The proportion of the population aged 0–14	0–14	A total population divided by population aged 0–14
		The proportion of the population aged above 65	Above 65	A total population divided by population aged above 65
		The proportion of the population with a higher education level	HD	A total population divided by population with an associate degree or higher

**Table 3:** Descriptive statistics of the variables.

Variable	Obs ^1^	Mean	Std. Dev. ^2^	Min. ^3^	Max. ^4^	Units
OV ^5^	372	7.76	11.68	0.17	87.06	Times/population
IV	372	0.19	0.27	0.19	14.733	Times/population
ED	372	83.32	128.21	1.16	1078.45	Amount/population
GE	372	0.06	0.10	0.00	0.89	Amount/population
PM2.5	372	3.58	0.51	1.94	4.44	μg/m^3^
SD	372	4.04	7.35	0.02	68.79	Amount/population
HWD	372	0.80	0.20	0.10	1.00	%
GDP	372	10.42	0.60	8.66	11.77	Yuan
TP	372	17.22	1.35	12.61	21.24	Person
PUP	372	0.53	0.14	0.23	0.90	%
0–14	372	0.17	0.04	0.07	0.27	%
65	372	0.09	0.02	0.04	0.14	%
HD	372	0.12	0.07	0.00	0.47	%

^1^ Obs = observations. ^2^ Std. Dev. = standard deviation. ^3^ Min. = minimum. ^4^ Max. = maximum. ^5^ OV: outpatient visits per capita; IV: inpatient visits per capital; ED: effluent discharge; GE: gas emission; SD: solid discharge; PM2.5: average PM2.5 concentration; HWD: household waste disposal; GDP: gross domestic product per capita; TP: total population; PUP: the proportion of the urban population; 0–14: the proportion of the population aged 0–14; 65: the proportion of the population aged above 65; HD: the proportion of the population with a higher education level.

**Table 4:** Global Moran’s I based on Matrix 1.

Year	Effluent Discharge	Waste Gas Emission	Solid Discharge	PM2.5 Concentration	Household Waste Disposal
2006	0.198	0.235 *^,1,2^	0.277 **	0.499 ***	0.110
2007	0.196	0.237 *	0.264 *	0.504 ***	0.277 **
2008	0.201 *	0.215 *	0.277 **	0.456 ***	0.365 ***
2009	0.208 *	0.205 *	0.288 **	0.453 ***	0.316 **
2010	0.245 *	0.171	0.244 *	0.425 ***	0.206 *
2011	0.236 *	0.240 *	0.236 *	0.498 ***	0.080
2012	0.252 *	0.221 *	0.226 *	0.446 ***	0.188
2013	0.270 *	0.168	0.218 *	0.498 ***	0.216 *
2014	0.246 *	0.242 *	0.261 **	0.416 ***	0.271 **
2015	0.234 *	0.269 *	0.281 *	0.492 ***	0.264 **
2016	0.257 *	0.148	0.247 *	0.507 ***	0.208 *
2017	0.269 *	0.088	0.281 **	0.452 ***	0.279 **

**^1^** *** *p* < 0.01, ** *p* < 0.05, * *p* < 0.1. **^2^** bold numbers represent significant coefficients.

**Table 5:** Empirical results of the spatial panel model.

Variable	Ln (OV) ^2^			Ln (IV)		
	Matrix One	Matrix Two	Matrix Three	Matrix One	Matrix Two	Matrix Three
Ln (GE)	0.195 * ^1,3^ **(2.14)**	0.137 (1.71)	0.350 *** **(3.93)**	0.017 (0.61)	0.041 (1.39)	−0.041 (−1.52)
Ln (ED)	0.575 *** **(5.12)**	0.775 *** **(9.87)**	0.373 ** **(2.63)**	0.114 ** **(2.90)**	0.036 (0.77)	0.027 (0.62)
Ln (SD)	0.126 * **(2.13)**	0.132 ** **(2.33)**	−0.013 (−0.21)	−0.007 (−0.34)	−0.020 (−1.01)	0.013 (0.70)
Ln (PM2.5)	−0.004 (0.330)	−0.002 (−0.73)	−0.001 (−0.15)	0.003 (1.50)	−0.002 (−1.03)	−0.001 (−0.64)
HWD	0.005 ** **(2.75)**	0.003 * **(1.90)**	0.005 * **(2.50)**	0.001 ** **(2.61)**	0.002 *** **(3.78)**	0.001 * **(2.13)**
Ln (TP)	0.652 *** **(5.21)**	0.257 *** **(3.44)**	0.788 *** **(4.72)**	0.868 *** **(14.08)**	−0.004 (−0.14)	1.017 *** **(17.89)**
PUP	2.007 ** **(2.80)**	1.614 ** **(2.34)**	3.862 *** **(3.51)**	0.891 ** **(2.75)**	1.176 *** **(3.36)**	0.851 ** **(2.71)**
0–14	3.160 * **(1.99)**	3.436 * **(2.18)**	4.505 * **(2.46)**	1.023 * **(1.97)**	0.588 (1.01)	1.021 (1.76)
Above 65	0.103 (0.04)	4.766 * **(2.08)**	4.646 (1.82)	3.154 *** **(4.04)**	1.408 (1.81)	3.263 *** **(4.31)**
Ln (GDP)	−0.202 (−1.20)	0.452 ** **(3.02)**	0.091 (−1.55)	0.263 *** **(3.82)**	0.559 *** (9.44)	0.448 *** **(6.55)**
HD	−1.047 (−1.21)	−0.868 (−1.06)	1.683 (−1.55)	0.048 (0.16)	0.524 (1.57)	−0.179 (−0.54)
W × Ln (GE)	0.251 (1.79)	−0.051 (−0.30)	0.146 (0.98)	−0.057 (−1.49)	−0.115 * **(−2.21)**	−0.042 (−0.93)
W × Ln (ED)	0.229 (1.64)	0.888 *** **(4.16)**	0.207 (0.69)	0.037 (0.60)	0.085 (0.53)	−0.029 (−0.29)
W × Ln (SD)	0.050 (0.49)	−0.223 (−0.88)	0.005 (0.03)	−0.024 (−0.76)	0.053 (0.61)	0.136 ** **(2.72)**
W × (PM2.5)	0.006 (0.99)	0.014 (1.68)	−0.005 (−0.66)	0.006 * **(2.25)**	0.003 (1.14)	0.000 (0.08)
W × HWD	−0.005 (−1.25)	−0.013 * **(−1.92)**	−0.031 *** **(−5.21)**	−0.000 (−0.22)	−0.005 * **(−1.99)**	−0.001 (−0.76)
W × Ln (TP)	−0.684 *** **(−5.37)**	−0.226 ** **(−2.82)**	−0.795 *** **(−4.74)**	−0.870 *** **(−14.11)**	0.008 (0.31)	−1.013 (−0.76)
W × PUP	−3.570 ** **(−2.77)**	−0.190 (−0.07)	−14.007 *** **(−5.80)**	−1.939 *** **(−3.70)**	−0.254 (−0.20)	−2.230 ** **(−2.75)**
W × 0–14	2.324 (0.94)	2.131 (0.46)	−7.500 (−1.89)	−2.371 ** **(−2.65)**	−4.752 (−1.80)	−0.802 (−0.56)
W × Above 65	−0.892 (−0.23)	−24.475 *** **(−3.54)**	−8.522 (−1.52)	−1.686 (−1.35)	−1.901 (−0.81)	−0.922 (−0.54)
W × Ln (GDP)	0.890 *** **(3.59)**	1.735 *** **(5.16)**	2.179 *** **(6.32)**	0.082 (0.84)	−0.315 (−1.49)	−0.044 (−0.39)
W × HD	1.976 (1.21)	−1.109 (−0.32)	7.338 ** **(2.66)**	−1.047 * **(−2.11)**	−2.098 (−1.85)	−1.613 * **(−1.98)**
ρ	0.5356 ***	0.699 ***	0.4977 ***	0.7454 ***	0.8215 ***	0.7493 ***
LLR	−171.8192	−139.5325	−166.9149	291.9434	247.1688	270.2405
R^2^	0.8225	0.8380	0.7712	0.9390	0.2128	0.9386
Obs	372	372	372	372	372	372

^**1**^ *** *p* < 0.01, ** *p* < 0.05, * *p* < 0.1. ^**2**^ OV: outpatient visits per capita; IV: inpatient visits per capital; ED: effluent discharge; GE: gas emission; SD: solid discharge; PM2.5: average PM2.5 concentration; HWD: household waste disposal; GDP: gross domestic product per capita; TP: total population; PUP: the proportion of the urban population; 0–14: the proportion of the population aged 0–14; 65: the proportion of the population aged above 65; HD: the proportion of the population with a higher education level. **^3^** bold numbers represent significant coefficients.

**Table 6:** Direct effects of the independent variables on healthcare.

Variable	Ln (OV) ^2^			Ln (IV)		
	Matrix One	Matrix Two	Matrix Three	Matrix One	Matrix Two	Matrix Three
Ln (GE)	0.167 (1.82)	0.150 (1.77)	0.350 ***^,1,3^ **(3.81)**	0.004 (0.906)	0.028 (0.83)	−0.057 (−1.89)
Ln (ED)	0.583 *** **(5.40)**	0.738 *** **(9.13)**	0.363 ** **(2.60)**	0.151 ** **(3.09)**	0.051 (0.77)	0.021 (0.40)
Ln (SD)	0.127 * **(2.05)**	0.161 * **(2.41)**	−0.010 (−0.15)	−0.016 (−0.70)	−0.013 (−0.49)	0.048 * **(1.92)**
Ln (PM2.5)	−0.003 (−0.90)	−0.001 (−0.29)	−0.001 (−0.32)	0.001 (0.83)	−0.001 (−0.83)	−0.001 (−0.69)
HWD	0.005 * **(2.45)**	0.002 (1.22)	0.002 (1.05)	0.002 * **(2.47)**	0.001 (1.74)	0.001 (1.52)
Ln (TP)	0.595 *** **(5.38)**	0.252 *** **(3.61)**	0.751 *** **(4.88)**	0.796 *** **(14.54)**	−0.003 (−0.12)	0.963 *** **(18.23)**
PUP	1.560 * **(2.06)**	1.690 * **(2.10)**	2.593 * **(2.38)**	0.478 (1.29)	1.304 *** **(3.20)**	0.489 (1.37)
0–14	3.858 * **(2.56)**	3.929 * **(2.55)**	3.885 * **(2.00)**	0.502 (0.83)	−0.106 (−0.13)	1.005 (1.31)
Above 65	0.114 (0.05)	2.093 (1.32)	4.179 (1.69)	3.432 *** **(4.23)**	1.305 (1.64)	3.682 *** **(4.43)**
Ln (GDP)	0.067 (0.43)	0.313 * **(2.06)**	0.328 (1.37)	0.352 *** **(5.49)**	0.585 *** **(8.66)**	0.515 *** **(6.47)**
HD	−0.781 (−0.84)	−1.013 (−1.11)	−1.020 (−0.95)	−0.283 (−0.78)	0.217 (0.52)	−0.591 (−1.51)

^**1**^ *** *p* < 0.01, ** *p* < 0.05, * *p* < 0.1. ^**2**^ OV: outpatient visits per capita; IV: inpatient visits per capital; ED: effluent discharge; GE: gas emission; SD: solid discharge; PM2.5: average PM2.5 concentration; HWD: household waste disposal; GDP: gross domestic product per capita; TP: total population; PUP: the proportion of the urban population; 0–14: the proportion of the population aged 0–14; 65: the proportion of the population aged above 65; HD: the proportion of the population with a higher education level. **^3^** bold numbers represent significant coefficients.

**Table 7:** Spill-over effects of the independent variables on healthcare.

Variable	Ln (OV) ^2^			Ln (IV)		
	Matrix One	Matrix Two	Matrix Three	Matrix One	Matrix Two	Matrix Three
Ln (GE)	0.288 (1.17)	−0.516 (−0.93)	−0.064 (−0.24)	−0.162 (−1.36)	−0.460 (−1.46)	−0.279 (−1.76)
Ln (ED)	0.156 (0.70)	−1.100 (−1.48)	−0.060 (−0.11)	0.429 (1.85)	0.556 (0.54)	−0.059 (−0.14)
Ln (SD)	−0.040 (−0.22)	−1.072 (−1.25)	0.002 (0.01)	−0.108 (−1.15)	0.193 (0.39)	0.559 **^,1,3^ **(2.66)**
Ln (PM2.5)	0.008 (−0.50)	0.040 (1.41)	−0.009 (−0.66)	−0.014 (−1.10)	0.010 (0.75)	−0.001 (−0.17)
HWD	−0.004 (−0.50)	−0.037 (−1.48)	−0.053 *** **(−3.83)**	0.003 (0.68)	−0.022 (−1.33)	−0.001 (−0.09)
Ln (TP)	−0.663 *** **(−5.54)**	−0.132 (−1.05)	−0.767 *** **(−4.83)**	−0.801 *** **(−13.85)**	0.036 (0.59)	−0.947 *** **(−15.97)**
PUP	−4.942 (−1.91)	3.227 (0.760)	−22.985 *** **(−5.23)**	−4.592 ** **(−2.56)**	4.064 (0.55)	−6.076 * **(−2.13)**
0–14	7.582 (1.54)	14.440 (0.82)	−10.111 (−1.31)	−6.135 * **(−1.90)**	−22.906 (−1.54)	0.012 (0.00)
Above 65	−1.702 (−0.25)	−9.276 ** **(−2.57)**	−12.119 (−1.20)	2.408 (0.60)	−4.986 (−0.40)	5.717 (0.87)
Ln (GDP)	1.539 *** **(−5.54)**	4.654 ** **(3.15)**	4.179 *** **(5.60)**	1.002 *** **(3.96)**	0.854 (0.70)	1.085 * **(2.48)**
HD	2.784 (0.82)	−5.951 (−0.49)	12.582 * **(2.43)**	−3.659 (−1.90)	−9.447 (−1.22)	−6.600 * **(−1.95)**

^**1**^ *** *p* < 0.01, ** *p* < 0.05, * *p* < 0.1. ^**2**^ OV: outpatient visits per capita; IV: inpatient visits per capital; ED: effluent discharge; GE: gas emission; SD: solid discharge; PM2.5: average PM2.5 concentration; HWD: household waste disposal; GDP: gross domestic product per capita; TP: total population; PUP: the proportion of the urban population; 0–14: the proportion of the population aged 0–14; 65: the proportion of the population aged above 65; HD: the proportion of the population with a higher education level. **^3^** bold numbers represent significant coefficients.

## Data Availability

Data is contained within the article or supplementary material. The data presented in this study are available in [Table S1].

## Supplementary Materials

The following are available online at https://www.mdpi.com/1660-4601/18/4/1784/s1, Table S1: Summarized data of environmental variables and healthcare variables and control variables.
